# Addition of contrast in ultrasound screening for hepatocellular carcinoma

**DOI:** 10.1016/j.redii.2023.100039

**Published:** 2024-02-07

**Authors:** Kathryn McGillen, Nabeal Aljabban, Robert Wu, Benjamin Shin, Ian Schreibman, Franklin Luke, James Birkholz

**Affiliations:** a Penn State Health Milton S Hershey Medical Center, Department of Radiology, 500 University Drive, Hershey, PA 17033, USA; b Penn State Health Milton S Hershey Medical Center, Department of Medicine, 500 University Drive, Hershey, PA 17033, USA; c Rutgers Robert Wood Johnson Medical School, Department of Radiology, MEB #404, 1 Robert Wood Johnson Place, New Brunswick, NJ 08901, USA; d George Washington University Hospital, Department of Radiology, 900 23rd St NW 2nd Floor, Washington DC 20037, USA

**Keywords:** Contrast-enhanced ultrasound, Ultrasound, Hepatocellular carcinoma, Screening, Cirrhosis

## Abstract

**Objective:**

Screening ultrasound for hepatocellular carcinoma (HCC) identifies lesions which require further characterization by a contrast-enhanced exam to non-invasively diagnose HCC. While ultrasound is recommended in screening, some HCC can be occult on grayscale imaging. The purpose of this study was to determine if the addition of ultrasound contrast (sulfahexafluoride) to screening ultrasound for HCC can identify more HCC lesions than grayscale sonographic imaging alone.

**Methods:**

All HCC screening ultrasounds that also had contrast were evaluated in this retrospective study. Patients with a focal lesion seen only after administration of contrast (OAC) were noted, as well as any follow-up imaging or pathology results. Additional variables collected included patient demographics, cirrhosis type, and laboratory values.

**Results:**

230 unique patients were included, of which 160 had imaging or pathology follow-up. 18 of these patients had an OAC lesion, of which 17 had follow-up. Among these OACs, there was one LIRADS M lesion (1/18, 5.6 %) and one bland portal vein thrombus identified, which were both confirmed on follow-up imaging. All LIRADS 4 OAC lesions were downgraded. No additional HCC were identified on follow-up imaging or pathology of these patients.

**Conclusion:**

Addition of contrast to screening ultrasound did identify additional lesions, portal vein thrombus, and high grade malignancy. However, as the incidence of OAC lesions was low (7.8 %, 18/230) and most of the lesions were not malignant, addition of post contrast sweeps through the liver is of low value in the low to medium at-risk cirrhotic population in identifying occult HCC.

## Highlights

• Ultrasound screening for hepatocellular carcinoma (HCC) identifies lesions that require further characterization by a contrast-enhanced exam – CT, MRI, or contrast ultrasound (CEUS) to non-invasively diagnose HCC. Screening by ultrasound is recommended but some institutions utilize multi-phase CT or MRI to screen as ultrasound could miss isoechoic lesions.
• This study looked to determine if the addition of contrast to screening ultrasound for HCC can identify more lesions than grayscale ultrasound alone and if these OAC lesions (only-after-contrast) increase sensitivity of screening by ultrasound by diagnosing more malignancies.
• The rate of identification of OAC during the study was low, and most intermediate LIRADS lesions were downgraded (LIRADS 3 and 4). It was accurate in identifying one ultrasound-occult malignancy and a portal vein thrombus, which were otherwise isoechoic.
• Clinical impact.
• The addition of contrast to all screening US is low value, but may still be considered in very high risk patients in the cirrhotic at-risk population if there are contraindications to CT or MRI.

## Graphical abstract

**Figure:**
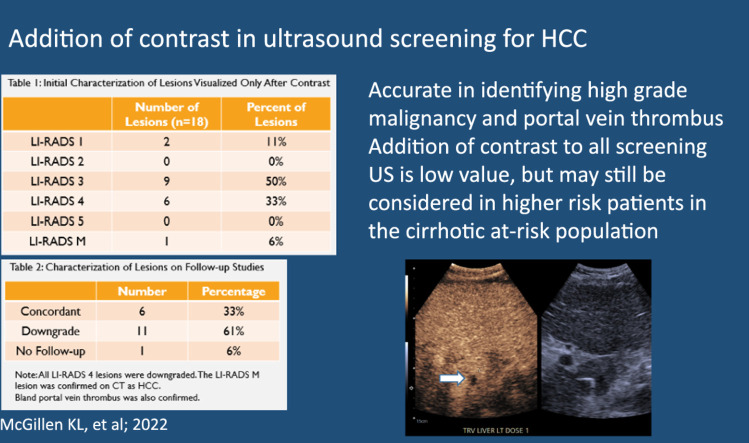

## 1 Introduction

Hepatocellular carcinoma (HCC) is the fourth leading cause of cancer-related mortality worldwide [1,2]. In patients with HCC, early diagnosis is paramount - five-year survival rates are greater than 50 % when detected early, while the detection after tumor symptoms develop is associated with a survival rate of only 10 % [3]. HCC detected at an earlier stage is amenable to liver transplantation or locoregional treatments such as trans-arterial chemoembolization or chemoradiation, radiation therapy, or ablation, which are associated with the best survival outcomes [4].

The gold standard non-invasive imaging screening options for HCC are multiphase computed tomography (CT) or magnetic resonance imaging (MRI) [5], however these may not be appropriate or approved by insurance for all at-risk patients. Limitations include allergic reactions to iodinated contrast used in CT, which are less common in CEUS, hesitancy of using iodinated contrast in patients with significantly impaired renal function [6], and non-diagnostic imaging in the presence of patient motion or breathing. In the screening population, access to accurate alternate imaging is paramount, of which ultrasound is readily available. Ultrasound is recommended as an initial screening modality in the American Association for the Study of Liver Disease (AASLD) guidelines, which recommend ultrasound every six months for high risk patients, with CT or MRI performed if ultrasound is inadequate [7,8].

During ultrasound screening, patients with any indeterminate or suspicious focal liver lesions require a return appointment for contrast-enhanced imaging to characterize the lesion and determine if it meets imaging diagnosis of HCC. Contrast enhanced ultrasound (CEUS) offers accurate [9,10,11] and non-invasive diagnosis of HCC for which Liver Imaging-Reporting and Data System (LIRADS) criteria can be applied [12], based on its arterial and washout characteristics.

Advantages of CEUS include its ability to allow for the real-time monitoring of enhancement and washout. Late phase arterial enhancement, which is important in non-invasive diagnosis of HCC, starts and ends rapidly, and if not optimally timed, may be missed on CT or MRI where imaging timepoints are fixed [13]. CEUS contrast agents remain intravascular and so allows for re-administration if initial characterization is inconclusive or characterization of additional lesions is necessary. However, both non-contrast and CEUS ultrasound can be limited by body habitus and degree of hepatic steatosis.

Screening ultrasound is generally performed without contrast. Ultrasound-occult HCC is a concern [8,14], which can be due to incomplete visualization of the liver parenchyma, background heterogeneity of the liver in cirrhotic patients, isoechoic lesions, or lack of a dedicated imaging protocol. Adding contrast when unenhanced ultrasound does not demonstrate any suspicious lesion has the potential to identify such occult lesions over background liver [15,16]. A screening protocol for HCC utilizing grayscale and CEUS has been described [17], and has been evaluated internationally using longer-acting Sonozoid (perfluorobutane), a contrast medium not currently Food and Drugs Administration (FDA)-approved in the United States [15,16,18]. Park et.al. found that the addition of Sonozoid reduced the false positive rate but did not improve detection rate of early-stage HCC [15]. A multi-institutional trial by Kudo et.al. however, found the addition of CEUS to be useful in identifying early HCC using a reinjection technique [16]. It is not known if Lumason® (sulfahexafluoride), a shorter acting agent could be effective in a more heterogeneous US population.

We postulate that lesions identified only after contrast administration (OAC) may be visible during post contrast sweeps through the liver - by their rapid enhancement, rapid washout, and/or on the delayed washout phase. This retrospective study looks at all OAC lesions found during screening ultrasound performed after sulfahexafluoride administration, to determine if the addition of ultrasound contrast in the setting of HCC screening by ultrasound has added value in identifying occult HCC.

## 2 Methods and materials

With institutional review board (IRB) approval and informed consent waiver, a retrospective study via chart review was conducted at our 546 bed semi-rural institution. All ultrasounds performed for HCC screening from January 2019 through December 2020 were identified. Subjects were included if they were (1) over the age of 18 and (2) they had a liver ultrasound looking for HCC and had contrast. Patients with a confirmatory follow-up test (multiphase CT, MRI, histologic, follow-up screening US) were included. Histologic confirmation included biopsy results, liver explantation, and autopsy results. Exclusion criteria included (1) CEUS ordered for a non-HCC liver lesion evaluation, (2) those who had CEUS for any other reason other than “screening for HCC.”

### 2.1 Imaging protocol

The screening protocol utilized consisted of grayscale images to evaluate for focal liver lesion or portal vein thrombus, as well as cine sweeps. Post contrast sweeps were performed from 0 to 1 min and from 3 to 4 min to look for enhancement and washout (early and delayed). Contrast microbubbles were then burst, and a second dose through the remaining liver lobe was performed similarly [17]. Images were acquired on Siemens or GE ultrasound units. Amount of sulfahexafluoride contrast was dependent on the machine used, with less required with newer software (1 mL + 5 mL saline flush for newer machines, and 2.4 mL + 5 mL saline flush for older machines).

### 2.2 Data collection

Variables collected include patient demographics (age, gender, height and weight, type of cirrhosis), information regarding the CEUS scan (enhancement and washout characteristics, LIRADS scores when applicable, portal vein patency, and OAC lesions), laboratory values (alpha-fetoprotein levels, MELD), and follow-up imaging or pathology results/concordance. Two fellowship-trained abdominal radiologists retrospectively reviewed the report and images for OAC (>10 years, and 8 years of experience respectively). These readers checked LIRADS designations and if not present, assigned them based on imaging criteria.

## 3 Results

### 3.1 Demographics

A total of 230 HCC screening ultrasounds using contrast were performed during the time period. Of these, 62.6 % (144/230) were men. Mean age at time of CEUS was 59.6 years (range of 20–85). Additional demographics are detailed in Table 1.

**Table 1:** Demographics of included patients.

	Patients
Characteristic	(*N* = 230)
**Age (mean ± SD)**	60 ± 13
**Gender (M/F)**	144/86
**Ethnicity**	
American Indian/Alaska native	0
Asian	16
Native Hawaiian/other Pacific Islander	0
Black or African American	13
White/Caucasian	180
More than one race	3
Unknown	18
Height	170 ± 10
Weight	91 ± 25
BMI	31 ± 7
**Cirrhosis Etiology**	
Hepatitis B	19
Hepatitis C	45
NASH vs. NAFLD	84
Alcohol	52
Cardiogenic	12
Cryptogenic	6
Other	41
**AFP baseline**	4.4 ± 7.2
**MELDNa baseline**	11 ± 6

### 3.2 Follow-up

Of the 230 patients, 160 had dedicated follow-up imaging of CT, MRI, or ultrasound (69.6 %). Eighteen OAC lesions were identified, of which 17 had follow-up (94.4 %).

### 3.3 Only after contrast lesions

**Fig. 1:**
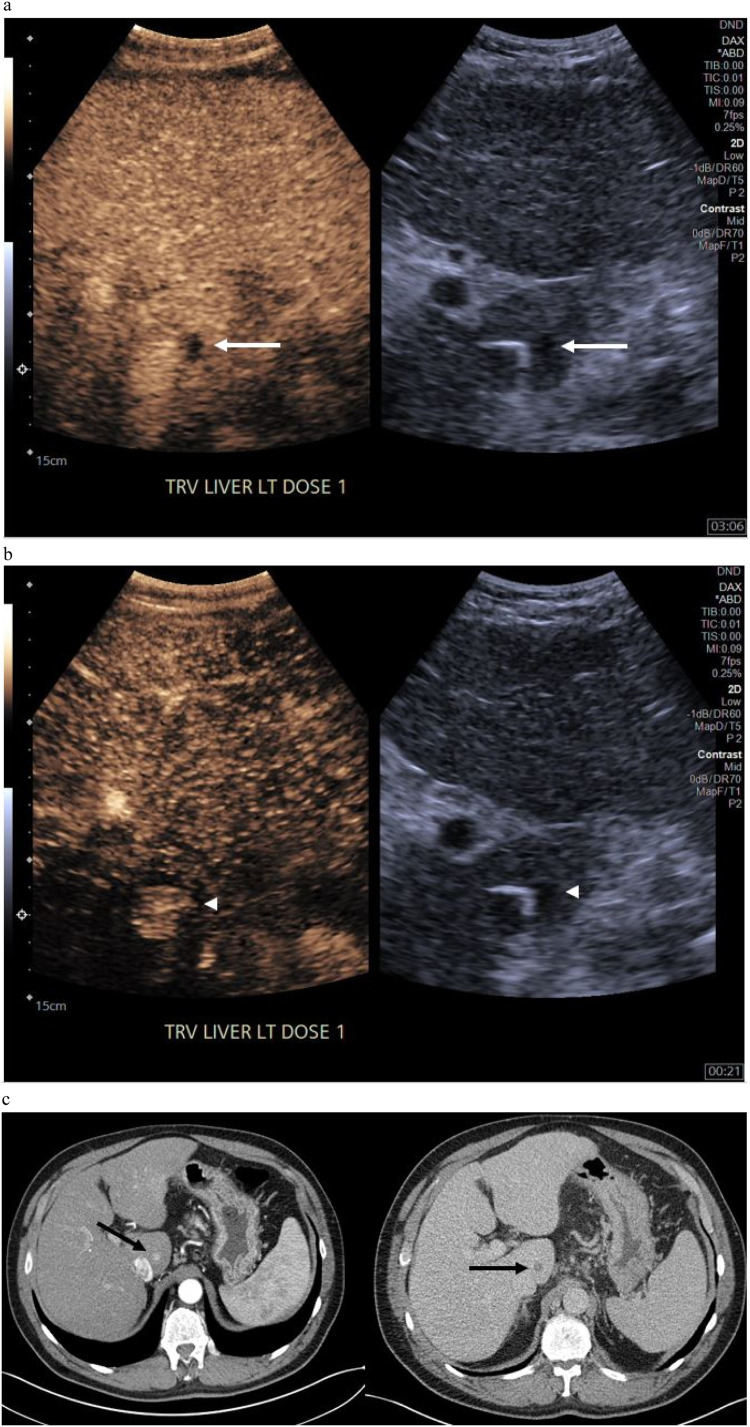
LIRADS-M OAC lesion – identified only after contrast administration and first recognized on delayed phase (a) white arrow. Arterial phase, the lesion was seen in retrospect when searching cine clips specifically for it (b), white arrow head. (c) Arterial and delayed phases show corresponding lesion on CT, black arrows.

Eighteen lesions were identified only after contrast administration (18/230, 7.8 %). Of these, the average lesion size was 1.3 cm (range 0.4 – 3.6 cm) compared to the entire cohort average lesion size of 1.8 cm (range 0.5 – 5.1 cm). LIRADS categories are summarized in Table 2 for OAC lesions. There were 8 lesions that had arterial enhancement only, 6 with mild delayed washout, 1 with early or marked washout only, and 2 with both enhancement and washout (one with marked washout, and one with late/mild washout). One lesion had no enhancement on any phase and was diagnosed as a small cyst that was only clearly apparent after contrast over background liver heterogeneity. LIRADS 3 designation was the most common (focal arterial hyperenhancement only or delayed mild washout only) at 50 % of patients (9/18). Six LIRADS 4 were identified, 0 LIRADS 5 lesions, and 1 LIRADS M. The LIRADS M was confirmed on subsequent 4 phase CT (1/18 OAC, 56 %, and 1/230 cohort, 0.4 %). The LIRADS M lesion was initially recognized on CEUS washout phase, and measured 0.8 cm (Fig. 1).

**Table 2:** Characterization of lesions visualized only after ultrasound contrast administration.

	Number of lesions (*n* = 18)	Percent of lesions	Concordant on follow-up
LI-RADS 1	2	11 %	Yes (2/2)
LI-RADS 2	0	0 %	Not applicable
LI-RADS 3	9	50 %	3/9, 1 lost to follow-up
LI-RADS 4	6	33 %	No (6/6)
LI-RADS 5	0	0 %	Not applicable
LI-RADS M	1	6 %	Yes (1/1)

**Fig. 2:**
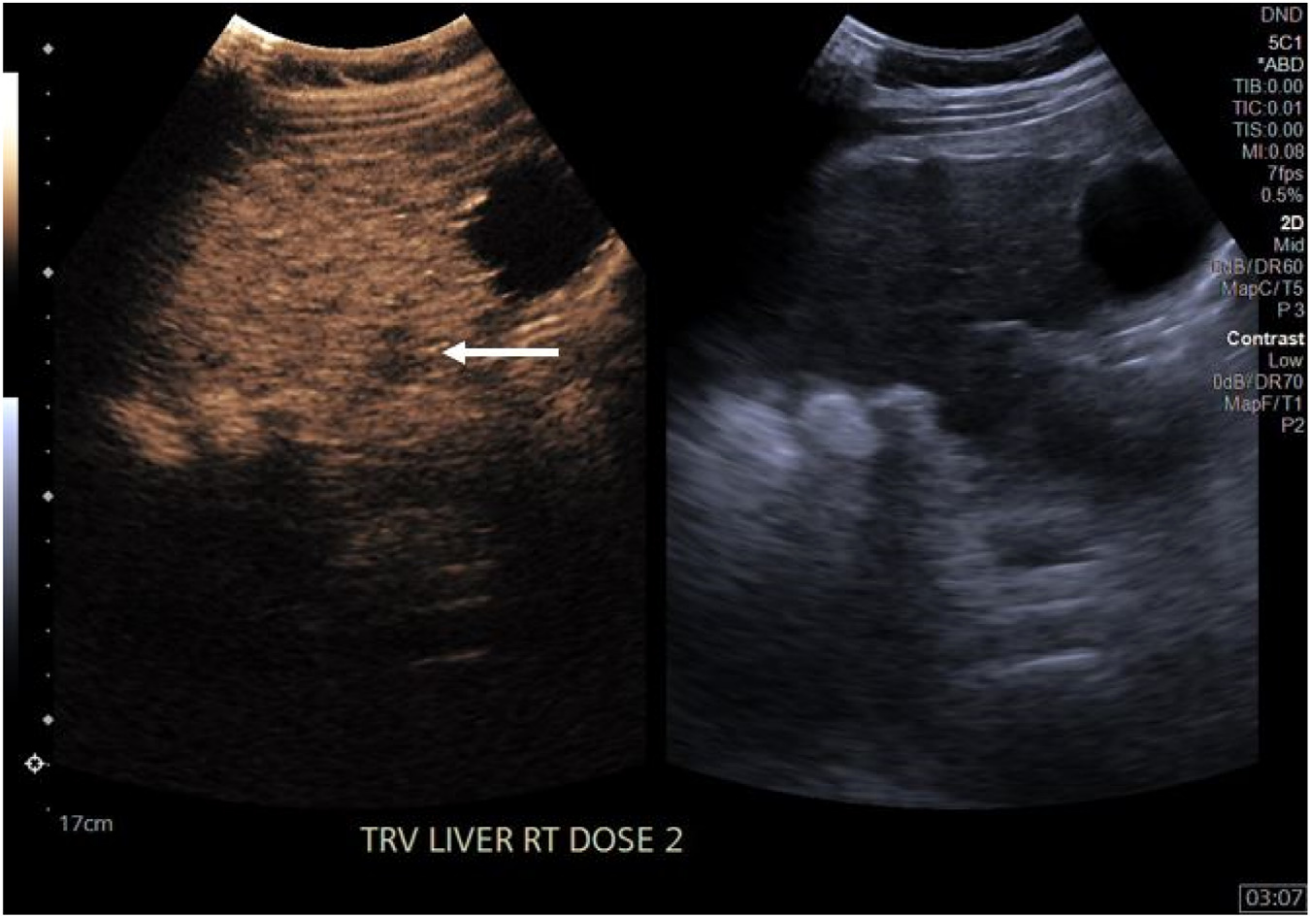
Downgraded LIRADS 3 lesion (white arrow). Lesion is seen only as washout on delayed phase, no correlate on arterial images or on 14 months of follow-up imaging.

**Fig. 3:**
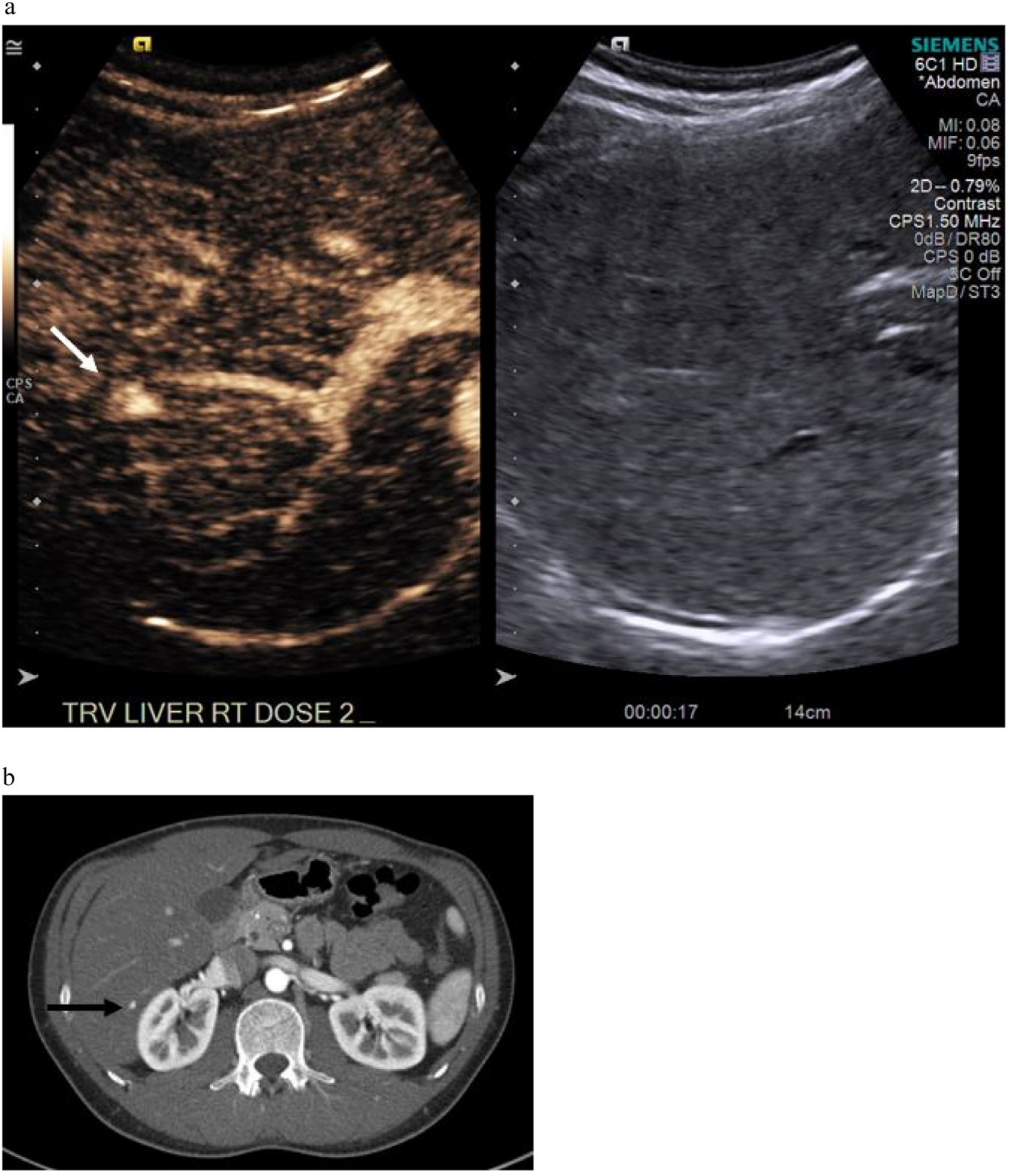
LIRADS 4 lesion that was downgraded on follow-up CT. A 1.1 cm lesion is seen on during arterial phase sweeps (a) denoted by white arrow. No washout on CEUS (not shown), and downgraded to LIRADS 3 on CT. (b) Arterial phase CT imaging shows the correlate lesion (black arrow), which was stable on 1.5 years of subsequent follow-up.

Six of the 18 lesions had concordant LIRADS scores (33 %) on follow-up cross-sectional imaging. Both LIRADS 1 lesions were concordant, as was the LIRADS M. Three of the nine LIRADS 3 lesions were also identified as LIRADS 3 on follow-up imaging. 11 lesion categories were downgraded (examples in Fig. 2, Fig. 3) including all LIRADS 4 lesions (61 %), while 1 had no follow-up (LIRADS 3). Review of the medical record indicated that this patient with no follow up imaging or pathology had not expired or been diagnosed with malignancy in the 2 years after their CEUS.

The mean AFP when available for patients with OAC was 4.5 with a mean MELD of 12.6, similar to the entire cohort. BMI of the OAC group was 35.1, higher than the entire group at 31. Mean age of 60 years, 61.1 % male (11/18) for OAC, was similar to the entire cohort.

One bland portal vein thrombus (lack of enhancement in the portal vein) was identified at CEUS and was confirmed on follow-up CT imaging.

No complications or allergic reactions were documented during the study.

## 4 Discussion

No high grade LIRADS lesions were missed in the 160 patients who had follow-up imaging, further supporting the use of ultrasound as a screening modality for HCC per AASLD guidelines [8]. The addition of contrast with sweeps through the liver to search for occult HCC did identify one malignancy that otherwise would have been missed on unenhanced grayscale screening alone.

Of the 18 OAC lesions identified, only a single lesion was ultimately confirmed as malignant (Fig. 1). Our low rate of occult malignancy detection after contrast administration confirms findings by Park et al. [15], who used perflurobutane, which is not currently FDA-approved in the US, but also found that the addition of contrast during sonographic HCC screening was not additive in their specific patient population, which consisted predominantly of hepatitis B positive patients. In our study, the patient population was more heterogeneous, led by fatty liver disease (36 %), followed closely by alcohol and hepatitis C related cirrhosis (Table 1), but our results nonetheless have similar findings.

Interestingly, the malignant lesion was identified during the delayed phase due to its marked washout (Fig. 1). The arterial enhancement was only appreciated after a review of cine clips specifically seeking out the lesion. During CEUS, this characteristic of delayed washout is of particular use in identifying LIRADS M lesions during post-enhancement screening imaging sweeps as washout last longer than the rapid arterial phase. However, LIRADS 5 HCC are defined by mild washout, a characteristic which can be challenging to identify on sweeps if there is significant residual background enhancement heterogeneity.

Our screening patient population consisted mostly of at-risk patients in the low and moderate HCC risk categories, which was supported by the cohort AFP and MELD scores. The MELD scores of the 18 patients with an OAC were similar to the overall cohort (12.6 versus 11, respectively), so it may not be surprising that most of the OAC enhancing lesions were not malignant. The lack of upgraded suspicious lesions or unidentified HCC lesions on follow-up may also support this. It is also possible that the small size of lesions could be a factor. The average lesion size for all patients was 1.8 cm, and 1.3 cm in the OAC category, with half of the OAC lesions measuring sub-centimeter. Accuracy of CEUS in characterizing smaller lesions is promising but is not yet widely established [19,20].

The retrospective nature of this study is an inherent limitation given reliance upon follow-up imaging and pathology availability. Our follow-up rate was fairly high, at nearly 70 % of patients and nearly all of the OAC lesions. The relatively low number of lesions seen only after contrast is a significant limitation, particularly as only 1 lesion of the 18 identified was malignant. A greater sample size of lesions visualized only after contrast would be needed to evaluate for any benefit in screening for very high risk patients.

In our patient population, mostly comprised of low to moderate risk patients in the setting of underlying fatty liver disease, an HCC screening program utilizing contrast enhanced ultrasound sweeps with bilobar imaging for isoechoic or occult HCC has proven to be of low value, since the incidence of occult OAC lesions is low (7.8 %) and the overall rate of malignancy in these even lower (0.04 %). Additional studies looking specifically at the highest risk cirrhotic population may show benefit for the addition of post contrast sweeps for US HCC screening program if the patient cannot otherwise tolerate CT or MRI due to other factors.

## Ethical statements

Statements and Declarations

Dr McGillen is compensated for lectures at MRIOnline.com

This retrospective study was approved by the IRB at our institution. Informed waiver of consent was waived by the IRB.

All authors contributed significantly to this manuscript. KM, NA, and JB contributed to data collection. KM, RW, BS, IS, and FL contributed to writing and editing. KM, FL, JB, and BS contributed to conception of the project.

## Declaration of competing interest

The other co-authors have no competing interests to declare.
